# *sqt-3(sc63)* is an alternative CRISPR/Cas9 co-conversion marker in *Caenorhabditis elegans*

**DOI:** 10.17912/micropub.biology.001467

**Published:** 2025-02-12

**Authors:** Isa Özdemir, Florian A. Steiner

**Affiliations:** 1 Department of Molecular and Cellular Biology, Section of Biology, Faculty of Sciences, University of Geneva, Geneva, Switzerland

## Abstract

The identification of genome-edited individuals by CRISPR/Cas9 in *Caenorhabditis elegans* often relies on the introduction of a second mutation with a visible phenotype. Popular targets for such co-conversion are *dpy-10* and *sqt-1* , both located on chromosome II. In this study, we introduce *sqt-3 ( sc63 )* *V* as an alternative CRISPR/Cas9 co-conversion marker to facilitate the generation and detection of genome edits in *C. elegans* . Its location on chromosome V makes *sqt-3* a desirable alternative to *dpy-10* and *sqt-1* when editing targets on chromosome II. Additionally, the conserved nature of *sqt-3* provides a potential applicability in other nematode species.

**Figure 1. The use of sqt-3(sc63) as a CRISPR co-conversion strategy in C. elegans:**
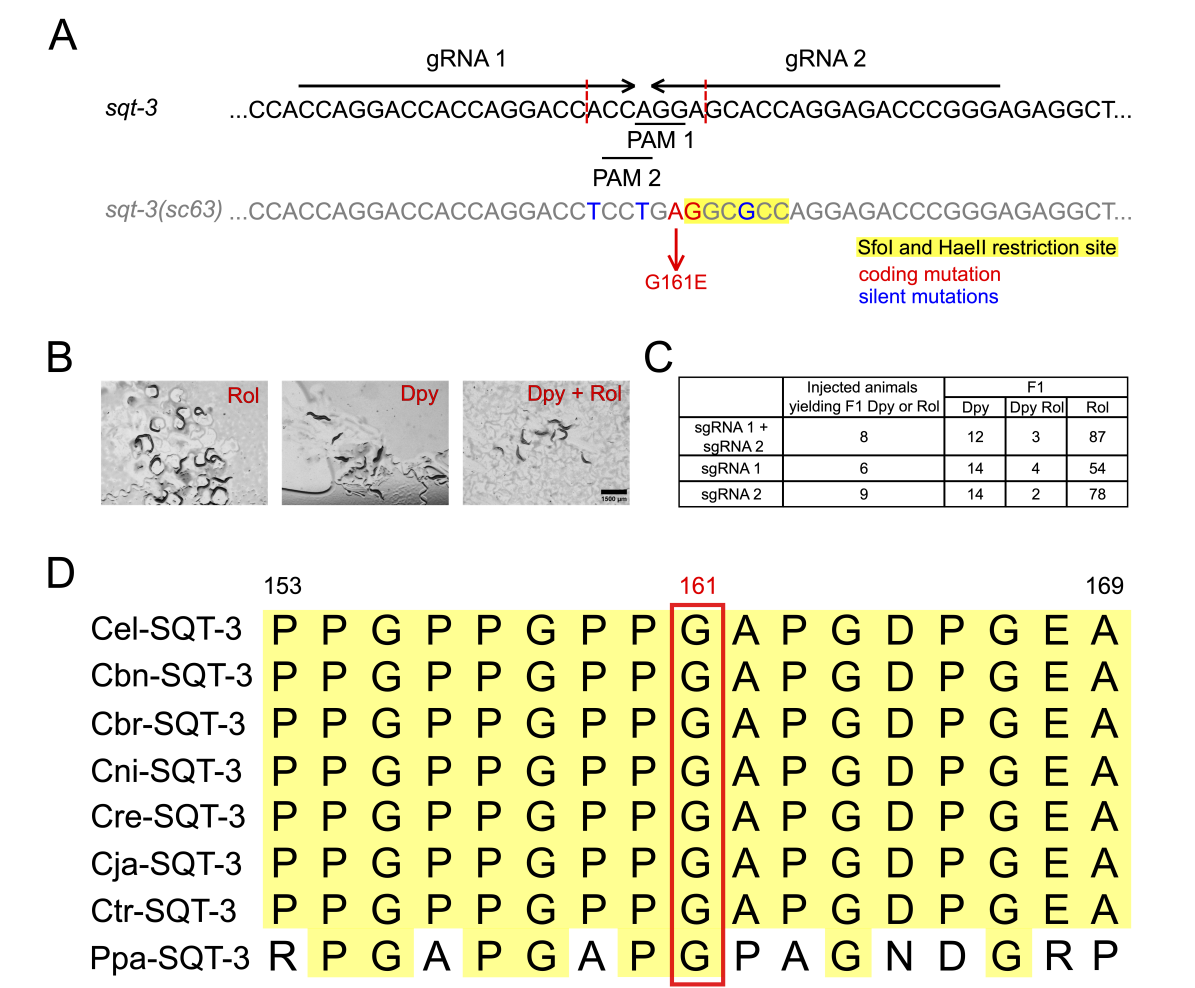
**(A)** Schematic representation of the *sqt-3* coding strand from amino acid residues 153 to 169, with the wild-type sequence on top and the edited sequence at the bottom. The *sc63* mutation (G161E) is indicated in red. The gRNA sequences are marked with black arrows, and the adjacent PAM sequences are underlined. Additional silent mutations introduced by the single-stranded DNA repair template create *SfoI* or *HaeII* restriction sites for ease of screening, which are highlighted with a yellow background. Silent mutations introduced to disrupt gRNA complementarity and prevent re-editing are shown in blue. The expected Cas9 cleavage sites are marked by vertical red dashed lines. **(B)** Representative images of Roller (left), Dumpy (middle), and Dumpy/Roller (right) phenotypes in F1 adult progeny of injected animals grown at 25°C. Individuals with the corresponding phenotype were placed in proximity to each other for imaging. Images were captured using a Leica DFC3000 G camera with a 20x objective. Scale bar: 1500 µm. **(C)** Summary of editing efficiency with each gRNA. About 20 animals were injected for each condition, resulting in 6–9 individuals producing edited F1 offspring at 25°C. **(D)** Alignment of SQT-3 amino acid residues 153–169 of *C. elegans* (Cel) to the homologous proteins in *C. brenneri* (Cbn), *C. briggsae* (Cbr), *C. nigoni* (Cni), *C. remanei* (Cre), *C. japonica* (Cja), *C. tropicalis* (Ctr), and *P. pacificus* (Ppa). Protein alignments were generated using SnapGene with default settings. The *C. elegans* residue G161 mutated in *sc63* is highlighted with a red box.

## Description

CRISPR/Cas9 genome editing has revolutionised genetic studies in biomedical research, including the model organism *C. elegans* (Friedland et al., 2013) . Cas9 is a double stranded DNA endonuclease that generates breaks upon the recognition of the target DNA locus through a protospacer adjacent motif (PAM) and a guide RNA (gRNA) that is complementary to the target sequence (Jinek et al., 2012; Liao et al., 2024) . The Cas9-induced double-stranded breaks are repaired by either error prone nonhomologous end joining (NHEJ) or, less frequently, homologous recombination (HR) using a single- or double-stranded donor DNA (repair template). In genome-editing, these repair templates typically include the desired edits, insertions or deletions flanked by 30–50 nucleotide arms with homology to the Cas9 target site.

Although CRISPR/Cas9 is a highly versatile tool to generate targeted mutations with base-pair precision, the editing efficiency is variable, and obtaining the desired edits often requires extensive screening and genotyping effort. To reduce this effort, co-editing strategies were developed for *C. elegans* (Arribere et al., 2014) . The aim of co-editing is to target the gene of interest and a gene with a visible phenotype at the same time. Specific mutations in the *dpy-10* or *sqt-1* genes, both encoding members of the collagen gene family, have emerged as popular choices due to their visually distinguishable phenotypes. These marker mutations produce easily recognizable Roller (Rol) or Dumpy (Dpy) phenotypes in the F1 when one or both copies of DNA, respectively, have been edited. The presence of these phenotypes indicates successful genome editing in the individual at least at the co-edited locus, making it a candidate for also containing the edit at the desired target locus. However, both *dpy-10* and *sqt-1* are located in gene-rich regions on chromosome II, limiting their utility when targeting genetically linked loci on the same chromosome. In such cases, separating the desired genome edit from the genetically linked marker mutations can be labor-intensive and time-consuming. There are other co-conversion markers elsewhere in the genome, e.g. *unc-58* on chromosome X. However, loss-of-function alleles of *unc-58* are hard to distinguish from wildtype alleles (as is the case for another frequently used co-conversion marker, *rol-6* ). This hinders the selection against CRISPR-edited loss-of-function mutations, thus limiting the utility of the locus in repeated rounds of CRISPR experiments (Arribere et al., 2014) .

To address these limitations and improve the co-editing approach, we introduce *sqt-3* as an alternative co-editing marker. *sqt-3* encodes for a collagen and is located on chromosome V. The *sqt-3 ( sc63 )* allele contains a missense mutation that replaces glycine (G) at position 161 with glutamic acid (E) **( Figure 1A )** . This G161E mutation results in a Squat (Sqt) phenotype, meaning heterozygotes display a Rol phenotype, while homozygotes exhibit a Dpy phenotype. The phenotypes are temperature-sensitive and more pronounced at 25 ^o^ C **( Figure 1B )** , while at 15 ^o^ C we observed a Long (Lon) phenotype (Kusch & Edgar, 1986) . The phenotypes allow for phenotypic selection and counterselection of *sqt-3 ( sc63 )* mutants in the F1 and F2 generations at 20 ^o^ C and 25 ^o^ C, similar to the *dpy-10* and *sqt-1* co-editing markers (Cox et al., 1980) . Loss of function of *sqt-3* results in a visible Dpy phenotype (as is the case for *sqt-1* and *dpy-10* ), and *mut/null* and *null/null* can be distinguished from *mut* /+ and +/+, respectively. This allows the selection against loss-of-function mutations, the presence of which might prevent subsequent rounds of co-conversion.

To introduce the *sqt-3 [G161E]* mutation in co-editing experiments, we designed a single-stranded DNA repair template and two gRNAs targeting the *sqt-3* locus **( Figure 1A )** . These gRNAs exhibited editing efficiencies comparable to those reported for the widely used *dpy-10 ( cn64 )* allele (Arribere et al., 2014) **( Figure 1C )** . The introduction of silent mutations within the repair template creates restriction sites for *SfoI* or *HaeII* , facilitating verification of the successful co-edit **( Figure 1A )** .

The amino acid sequence of SQT-3 around G161 is fully conserved in other *Caenorhabditis* species, including *C. brenneri* , *C. briggsae* , *C. nigoni* , *C. remanei* , *C. japonica* , and *C. tropicalis* , and the G161 residue is also present in *P. pacificus* **( Figure 1D )** . This conservation suggests that the *sqt-3 ( sc63 )* mutation could serve as a versatile co-editing marker in other nematodes, expanding its utility beyond *C. elegans* .

In summary, *sqt-3 ( sc63 )* *V* represents a valuable and convenient addition to the toolkit of CRISPR/Cas9 co-editing markers for *C. elegans* . Its different chromosomal location and temperature-dependent penetrance make it a practical alternative when *dpy-10* or *sqt-1* markers are unsuitable. The conserved amino acid sequence of SQT-3 also makes it potentially applicable in nematode species beyond *C. elegans* .

## Methods

**Worm Strains and Maintenance:**

N2 (wildtype) strain was obtained from the *Caenorhabditis* Genetics Center, University of Minnesota. Strains were grown at 15°C, 20°C or 25 ^o^ C on NGM agar plates seeded with *E. coli * OP50 .

**CRISPR/Cas9 genome edits:**

Cas9 and gRNAs were injected into the gonads of N2 adult hermaphrodites in the form of plasmids as described in (Arribere et al., 2014) . gRNAs were cloned as described in (Arribere et al., 2014) , and listed in **Table 1** . The sequence of the repair oligonucleotide is given in **Table 2** . All oligonucleotides were ordered from Microsynth. The Cas9 worm expression plasmid (pDD162, #47549) and empty vector for gRNA cloning (pRB1017, #59936) were obtained from Addgene (Arribere et al., 2014; Dickinson et al., 2013) . The composition of the injection mix is listed in **Table 3** . A Leica DMIRE2 microscope and a Femtojet 4i (Eppendorf) were used for injections. F1 and F2 worms were screened for the desired phenotype under a standard dissecting microscope.

**Identification of the SQT-3 orthologues:**

The predicted SQT-3 protein sequences for *C. brenneri* , *C. briggsae* , *C. nigoni* , *C. remanei* , *C. japonica* , *C. tropicalis* , and *P. pacificus* were collected from Wormbase (WS294) based on nematode orthologs in the homology section.

The obtained sequences were aligned using Snapgene (version 8.0). Only the 8 aa upstream and 8 aa downstream of G161 in the alignment are shown in **Figure 1D .**

**Table 1. gRNA plasmids generated for this study**

**Table:** 

**Plasmids**	**Backbone**	**Insert**	**Description**
pIO57	pRB1017	CCAGGACCACCAGGACCACC	gRNA 1
pIO58	pRB1017	CCCGGGTCTCCTGGTGCTCC	gRNA 2

**Table 2. Oligonucleotides used in this study.**

**Table:** 

**Oligos**	**Sequences**	**Description**
oIO233	TCTTGCCAGGACCACCAGGACCACC	To clone gRNA 1 (pIO57)
oIO234	AAACGGTGGTCCTGGTGGTCCTGGC	
oIO235	TCTTGCCCGGGTCTCCTGGTGCTCC	To clone gRNA2 (pIO58)
oIO236	AAACGGAGCACCAGGAGACCCGGGC	
oIO237	CCACCATGCAAGCCATGCCCACAAGGACCACCAGGACCACCAGGACCTCCTGAAGCTCCAGGAGACCCGGGAGAGGCTGGAACCCCAGGACGCCCAGGGACCG	ssDNA (Repair template)
oIO263	GCCTACGGAGGACCAGAAG	To genotype and sequence the *sqt-3* locus
oIO264	CAAAACTCACTTTGGACAG	

**Table 3. Composition of the injection mix for the introduction of the *sqt-3 ( sc63 )* *V* mutation.**

**Table:** 

**Name**	**Description**	**Final concentration**
pDD162	Cas9 expressing plasmid	50 ng/µl
pIO57 and pIO58	gRNA expressing plasmids	25 ng/µl per gRNA when used together, 50ng/µl total
Alternative: pIO57 or pIO58	gRNA expressing plasmids	25 ng/µl per gRNA when used individually
oIO237	Single stranded repair template	500 nM
